# Supplementary figures and images for: Integration of full-length Iso-Seq, Illumina RNA-Seq, and flavor testing reveals potential differences in ripened fruits between two Passiflora edulis cultivars
Source: PeerJ. 2024 Sep 11;12:e17983. doi: 10.7717/peerj.17983 (PMC11401511; doi:10.7717/peerj.17983)

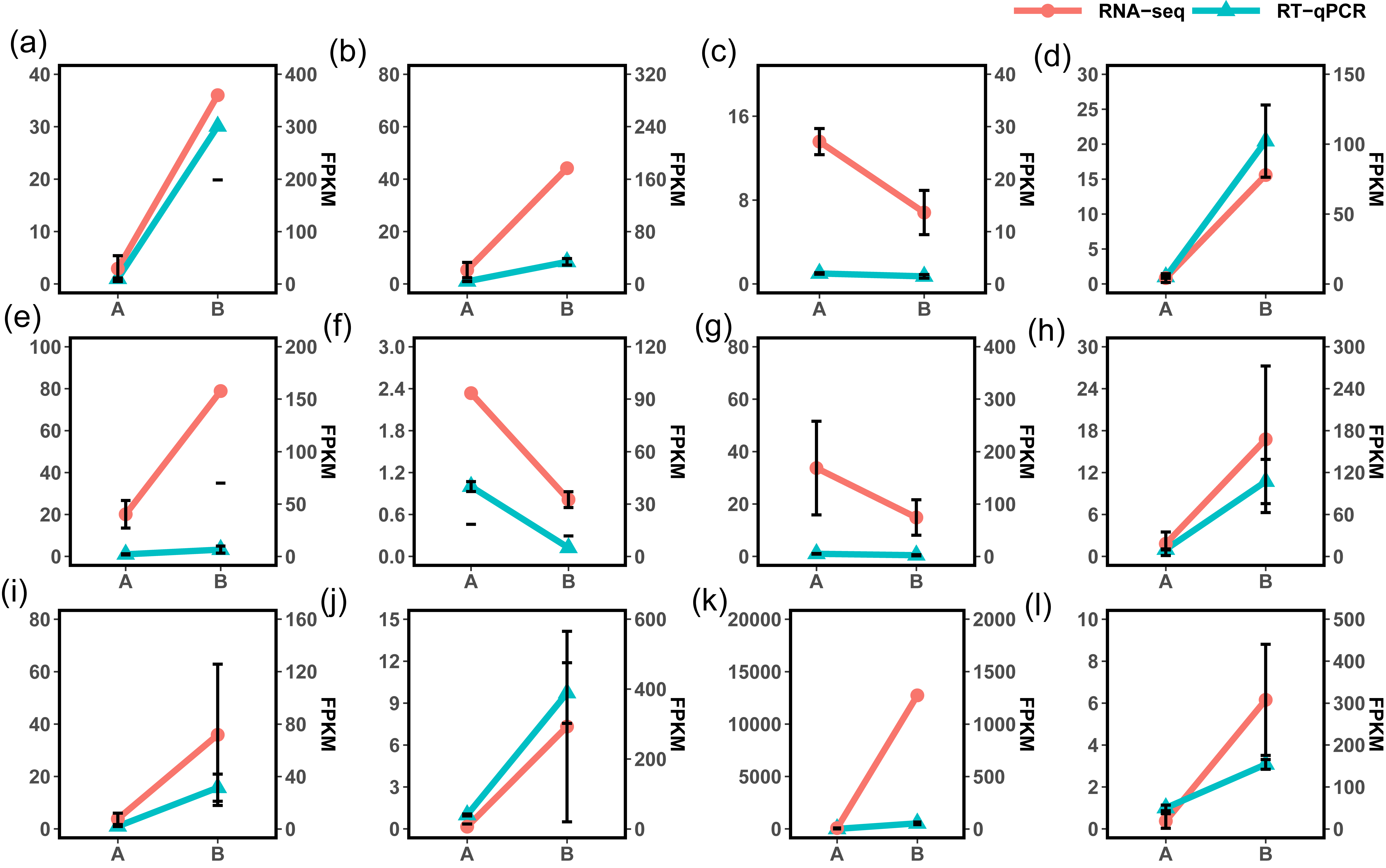

Supplement: Supplemental Information 1 — (a) ~ (i) were PCL_3727, PCL_5183, PCL_8874, PCL_16124, PCL_18357, PCL_19350PCL_18502, PCL_19539, PCL_20808, PCL_27524, PCL_30866, PCL_33444, respectively. [file peerj-12-17983-s001.png]
